# Self monitoring of blood glucose - a survey of diabetes UK members with type 2 diabetes who use SMBG

**DOI:** 10.1186/1756-0500-3-318

**Published:** 2010-11-22

**Authors:** Katharine D Barnard, Amanda J Young, Norman R Waugh

**Affiliations:** 1 NIHR Evaluation Trials and Studies Coordinating Centre, University of Southampton, Southampton Science Park, Chilworth, Southampton SO16 7NS UK; 2 Wessex Institute, University of Southampton, Southampton Science Park, Chilworth, Southampton SO16 7NS UK; 3 Department of Public Health, University of Aberdeen, Foresterhill, Aberdeen AB25 2ZD UK

## Abstract

**Background:**

Aim - to survey members of Diabetes UK who had Type 2 diabetes and who used self monitoring of blood glucose (SMBG), to elicit their views on its usefulness in the management of their diabetes, and how they used the results. A questionnaire was developed for the Diabetes UK website. The questionnaire was posted on the Diabetes UK website until over 500 people had responded. Questions asked users to specify the benefits gained from SMBG, and how these benefits were achieved. We carried out both quantitative analysis and a thematic analysis for the open ended free-text questions.

**Findings:**

554 participants completed the survey, of whom 289 (52.2%) were male. 20% of respondents were recently diagnosed (< 6 months). Frequency of SMBG varied, with 43% of participants testing between once and four times a day and 22% testing less than once a month or for occasional periods.

80% of respondents reported high satisfaction with SMBG, and reported feeling more 'in control' of their diabetes management using it. The most frequently reported use of SMBG was to make adjustments to food intake or confirm a hyperglycaemic episode.

Women were significantly more likely to report feelings of guilt or self-chastisement associated with out of range readings (p = < .001).

**Conclusion:**

SMBG was clearly of benefit to this group of confirmed users, who used the results to adjust diet, physical activity or medications. However many individuals (particularly women) reported feelings of anxiety and depression associated with its use.

## Background

A recent review of evidence on self monitoring of blood glucose (SMBG) in Type 2 diabetes, done to inform the deliberations of a Department of Health (England) working group on SMBG, found that the published evidence for effectiveness and cost-effectiveness was weak, and its value not proven [1]. However the working group had heard from Diabetes UK that many people with type 2 diabetes are convinced that SMBG is of value. The report of the working group is published http://www.diabetes.nhs.uk/document.php?o=1023[2]. Some individuals with Type 2 diabetes report the ability to self-monitor blood glucose levels to be empowering, enabling them to feel more 'in control' of their diabetes and able to react to readings quickly, rather than having to wait for their routine HbA1c test [3] Possible benefits of SMBG include immediate confirmation of hypoglycaemia or hyperglycaemia; an increase in motivation to stimulate greater self-care; and data with which patients or healthcare teams could adjust treatment regimens [3]. However, a recurring theme in the recent review by Clar and colleagues was that the results were often not used to adjust treatment.

Given the mismatch between the views of some patients, and the evidence from trials, it was decided to seek views from patients who valued SMBG, and in particular to find out how they used the data, and what changes they made.

It was anticipated that a highly selected group of people with type 2 diabetes who were SMBG users would respond, but rather than causing a problem of bias, this would provide information on why some people found SMBG valuable.

## Methods

A questionnaire was developed and piloted by seventeen volunteer service users and healthcare professionals prior to 'going live' on the Diabetes UK website. Questions were asked about duration of diabetes, frequency, uses and helpfulness of SMBG, about who provided information about how to use the meter and interpret results, what actions were taken in response to high or low blood glucose levels, and how participants felt when blood glucose results were out of target range. Forty questions were included with an estimated completion time of approximately 10-15 minutes. The questionnaire was posted on the Diabetes UK website from 15^th ^to 28^th ^January 2010. A copy of the questionnaire is attached as Appendix One.

Statistical analyses were performed using SPSS for Windows software (version 17). Categorical variables were analysed using descriptive and inferential statistics. Unless otherwise stated, the chi-square test was used for all comparisons of the categorical data (statistically significant at P < 0.05). If a 2 × 2 table was presented, the continuity for correction value was used and if any cell had an expected count less than 5 the Fisher's Exact Test was used. The free text responses were analysed using thematic analysis. The quality (including accuracy and completeness) of the thematic framework was independently checked by a second researcher. Any discrepancies were resolved by consensus.

For thematic coding and analyses, the quality check revealed an overall discrepancy of 5.1%. This figure represents the number of discrepancies/errors between the data entered by one researcher and an independent researcher reviewing the coding framework and consistency check of all data fields.

## Results

A total of 555 participants completed the survey. One participant was excluded as they did not have Type 2 diabetes, leaving 554 participants, of whom 289 (52.2%) were male. Of those who responded, 98.9% (n = 531) reported self-monitoring their blood glucose levels (n = 271; 98.2% men and n = 254; 99.6% women). None of the 1.1% of individuals who reported not self monitoring gave a reason for not doing so. Duration of diabetes details are provided in Table 1, with frequency of self-monitoring of blood glucose and timings of monitoring presented in Tables 2 and 3 respectively.

**Table 1:** Duration of Diabetes

	Male		Female		Total	
	*N*	%	*N*	%	*N*	%

0-6 months	57	19.8	54	20.5	111	20.1

Between 6 months and 1 year	36	12.5	20	7.6	56	10.1

Between 1 and 3 years	78	27.1	74	28.0	152	27.5

Between 3 and 5 years	38	13.3	43	16.3	81	14.7

More than 5 years	77	26.7	72	27.2	149	27.0

Don't know	1	0.3	0	0.0	1	0.2

Prefer not to say	1	0.3	1	0.4	2	0.4

Totals	288	100	264	100	552	100

**Table 2:** Frequency of SMBG

	Male		Female		Total	
**Frequency**	*N*	%	*N*	%	*N*	%

Once a day	33	12.2	38	15.0	71	13.5

Twice a day	29	10.7	32	12.6	61	11.6

Three times a day	25	9.2	22	8.7	47	9.0

Four times a day	16	5.9	19	7.5	35	6.7

More than four times a day	10	3.7	15	5.8	25	4.8

Once a week	28	10.3	15	5.8	43	8.2

More than once a week but not every day	52	19.2	39	15.4	91	17.3

Fortnightly	13	4.8	7	2.8	20	3.8

Less than once a month	6	2.2	4	1.6	10	1.9

Occasional periods *	54	19.9	60	23.6	114	21.7

Other	5	1.9	3	1.2	8	1.5

Totals	271	100	254	100	525	100

**Table 3:** Typically when do you test your bloods?

	Male		Female		Total	
**Frequency**	*N*	%	*N*	%	*N*	%

Before meals	178	65.7	174	68.5	352	67.0

2 hours after meals	122	45.0	122	48.0	244	46.5

Before going to bed	57	21.0	63	24.8	120	22.9

During episodes of illness	53	19.6	57	22.4	110	21.0

Before I set off to drive	14	5.2	16	6.3	30	5.7

If I feel hypoglycaemic	49	18.1	66	26.0	115	21.9

If I feel hyperglycaemic	68	25.1	98	38.6	166	31.6

Before periods of physical activity	12	4.4	19	7.5	31	5.9

During physical activity	5	1.9	4	1.6	9	1.7

After physical activity	25	9.2	27	10.6	52	9.9

A change to treatment being considered	19	7.0	17	6.7	36	6.9

Monitor effect of certain foods	74	27.3	88	34.7	162	30.9

Other	42	15.5	39	15.4	81	15.4

Totals	718	-	790	-	1508	-

* Respondents were able to tick more than one answer

### Characteristics of Respondents

95.8% (n = 531) of participants were on diet alone or oral glucose lowering drugs,, whilst 4.2% (n = 23) were using insulin. Of these, 98.9% (n = 525) used their own meter to test their blood glucose levels. The insulin users did not continue with the rest of the survey as they did not meet the inclusion criteria (i.e. Type 2 diabetes treated with lifestyle or oral medications).

The frequency of testing varied with the reasons for doing so, but there was wide variation within each group. Of the 195 who were testing to monitor control as they were newly diagnosed, 27.7% (n = 54) tested once or twice a day, with a further 16.4% (n = 32) testing four or more times a day. Amongst those testing to make adjustments to food intake, 24.1% monitored occasionally or less than once a month, but 25.1% (n = 72) tested once or twice a day. Amongst those testing to assess the impact of physical activity, 24.6% (n = 30) tested occasionally but over a third, i.e. 36.2% (n = 44) were testing three or more times a day.

Amongst those testing to confirm an episode of hypoglycaemia, 35.3% (n = 43) tested less than once a month or occasionally, but 42% (n = 51) tested one to more than four times a day, perhaps because they had episodes of hypoglycaemia. Similarly, amongst those testing to confirm hyperglycaemia, 25.6% (n = 72) did so less than once a month or occasionally but 42.9% (n = 121) tested between once to more than four times a day. Only 5.9% (n = 31) tested to alter their medication, whilst 15.5% (n = 81) tested to assess the effect of new medications.

43.1% of participants were testing their blood glucose levels between once a day and more than four times a day, compared with 22.4% (n = 124) who were testing less than once a month or for occasional periods. Their results did not differ systematically on the helpfulness question, nor were frequent user more anxious. It is not known whether this reflects healthcare professional advice in terms of testing regimen. Only 7.2% (n = 40, Table 4) of participants reported using SMBG to determine whether it is safe to drive.

**Table 4:** Uses of SMBG

	Male		Female		Total	
**Action**	*N*	%	*N*	%	*N*	%

Monitor my BG as I have recently been diagnosed with diabetes	100	36.9	95	37.4	195	37.1

Make adjustments to my food intake	140	51.7	146	57.5	286	54.5

When assessing the impact of physical activity	60	22.1	62	24.4	122	23.2

Confirm if I'm having a hypoglycaemic episode	50	18.5	72	28.4	122	23.2

Confirm if I'm having a hyperglycaemic episode	130	48.0	152	59.8	282	53.7

Alter my diabetes medication	22	8.1	9	3.5	31	5.9

Inform me about the effect of a new diabetes medication I've started	44	16.2	37	14.6	81	15.4

Inform me about my blood glucose levels during a period of illness	70	25.8	89	35.0	159	30.3

I don't use my blood glucose test results to do anything	13	4.8	10	3.9	23	4.4

Monitor my blood glucose levels as I am trying to conceive	N/A	N/A	3	1.2	3	0.6

Monitor my blood glucose levels as I'm pregnant	N/A	N/A	3	1.2	3	0.6

Determine whether it is safe to drive	23	8.5	17	6.7	40	7.6

Don't know	3	1.1	1	0.4	4	0.8

Total	658	-	696	-	1354	-

* Respondents were able to tick more than one answer; % figures are rounded

### Uses and Helpfulness of SMBG

Reasons for self-monitoring were varied as can be seen in Table 4 below. The extent to which participants felt SMBG was helpful is presented in Table 5 below.

**Table 5:** Helpfulness of SMBG (question 16)

1-10 Scale (1-extremely unhelpful, 10 very helpful)	Male		Female		Total	
	
	*N*	%	*N*	%	*N*	%
1	4	1.4	7	2.8	11	2.1

2	2	0.7	3	1.2	5	1.0

3	9	3.3	6	2.4	15	3.0

4	1	0.4	3	1.2	4	0.8

5	15	5.5	22	8.7	37	7.1

6	14	5.7	10	3.9	24	4.6

7	32	11.8	28	11.0	60	11.4

8	56	20.5	47	18.5	103	19.6

9	25	9.3	24	9.5	49	9.4

10	112	41.4	104	40.8	216	41.0

Total	270	100	254	100	524	100

* 524 respondents (94.6% of overall total)

Thus, 83.3% of male (n = 225) and 79.9% (n = 203) of female respondents scored 7 or above (81.7% total), indicating a high satisfaction with the usefulness of SMBG. This was as expected from a group who were expected to be mainly confirmed users.

Only five responses were received to the question 'if you circled 0-4, why don't you find SMBG helpful?' Three reported futility of testing e.g. "there is no point it changes nothing", "it's not helpful as you cannot do anything to bring you levels down ..."; one reported being demotivated i.e. "Whilst on medication, I found it dispiriting and demotivating to find that there was no significant reduction in levels over time".

There were no significant differences between participants reporting low helpfulness of SMBG (i.e. 0-4) and those reporting high helpfulness (i.e. 7-10) in terms of uses of SMBG, frequency of testing or duration of diabetes, but as expected the number of those reporting low usefulness was very low(n = 16).

The results did not differ statistically significantly when sub-group analysis was conducted for duration of diabetes, for male compared to female, or for frequent users compared with occasional users, expect for self chastisement issues being significantly higher for women than men.

### Responses to Out of Range Readings

Thematic analysis was conducted on free text responses to out of range readings. The results are detailed in Tables 6 and 7 below. Most patients responded to abnormal levels by diet and physical activity, with few adjusting medication, presumably because they could only do if their doctor changed their prescription. Responses to how participants felt when they experienced out of range blood glucose readings are detailed in Table 8 below.

**Table 6:** Free text responses to high blood glucose results (question 13)

Thematic Codes	Number of Responses
Adjust diet/eat less	175

Exercise	98

Doesn't happen/N/A	52

Do nothing	57

Test again later	45

Try to identify what caused it	38

Drink more fluids, particularly water	41

Unclear	31

Monitor food intake	33

Increase medication (oral anti-hyperglycaemic)	11

Consult or discuss with health care professional	31

Eat more chocolate	1

Total	613

* 140 (out of 473) respondents gave more than one response to this question

** The quality check revealed a 5.7% discrepancy between the two analysts?. These differences were resolved by consensus.

**Table 7:** Free text responses to low blood glucose result

Thematic Codes	Number of Responses
Eat/drink something	248

It doesn't happen/N/A	168

Increase SMBG frequency/retest later	27

Do nothing	18

Response unclear	18

Consult/note in diary to discuss with healthcare professional	9

Exercise	1

Increase starch intake	3

Total	492

* 34 (out of 460) respondents gave more than one response to this question

** The quality check revealed a 3.4% discrepancy between the two analysts. Differeneces were resolved by consensus

**Table 8:** How do you feel when your blood glucose is not as expected?

Thematic Codes	Number of Responses
Worried/anxious	135

In control/able to rationalize/determined to achieve better diabetes control/better informed	135

It doesn't happen/N/A	64

Frustrated/angry (with self and diabetes)/guilty	59

Disappointed	50

Not in control/low mood/upset/confused	68

Depressed	42

Physically unwell	16

Total	569

* 74 (out of 495) respondents gave more than one response to this question

* The quality check revealed a 4.2% discrepancy between the two reviewers. Differences were resolved by consensus

#### Do you have any concerns/worries about self monitoring of your blood glucose?

There were 525 responses to this question and of these, 89 (16.1%) expressed concerns or worries about self monitoring. Whilst only 16.1% reported having concerns/worries about SMBG on the questionnaire, the results from the free text questions was much higher, i.e. 27.3% (n = 135) of participants (out of the 495 who provided a response) reported anxiety and worry associated with blood glucose levels not being what they expected them to be. Furthermore, 8.5% (n = 42) reported feeling depressed, 13.7% (n = 68) not being in control/low mood/upset/confused and 11.9% (n = 59) felt frustrated/angry/guilty. Table 9 presents the actions taken by participants in response to an unexpected blood glucose reading.

**Table 9:** Actions Taken if blood glucose levels not as expected

Action	Male		Female		Total	
	*N*	%	*N*	%	*N*	%

I increased my medication	24	8.9	18	7.1	42	8.0

I reduced my carbohydrate for the same mealtime the next day	167	61.6	153	60.2	320	61.0

I did more physical activity than I would normally do	106	39.1	87	34.3	193	36.8

I contacted my diabetes healthcare team for advice	36	13.3	30	11.8	66	12.6

I didn't do anything	54	19.9	60	23.6	114	21.7

Other	21	7.8	17	6.7	38	7.2

Total	408	-	365	-	773	-

* Respondents were able to tick more than one answer

### Healthcare Professional Input

#### Who provided you with information about ...?

Most (74.7%, n = 211) participants reported that nurses provided information about how to use their meter, followed by 10.5% (n = 29) being informed by their doctor, 4.2% by a pharmacist and 11.0% reporting 'other'. Similarly, nurses suggested target blood glucose levels to 60.4% (n = 217) of participants, followed by doctors (35.1%; n = 126); a single pharmacist, and others (4.2%; n = 15). This pattern was repeated for information about how often to monitor blood glucose levels with nurses providing information to 65.0% (n = 208) of participants, doctors to 31.9% (n = 102), pharmacist to 0.6% (n = 2) and other to 2.5% (n = 8).

Nurses were the main source (67.5%) of information regarding what to do with the results, followed by, doctors 29.6% (n = 72), pharmacists 1.2% (n = 3) and others 1.6% (n = 4). Similarly, advice on what to do when readings were out of range information was provided by nurses 62.3% (n = 152), doctors 35.7% (n = 87), pharmacists 0.4% (n = 1) and other 1.6% (n = 4).

## Discussion

This group of confirmed users clearly value SMBG, and use it for self-management.

There was a good response to the survey with over 500 responses received within the two week window that the survey was live on the Diabetes UK website. This was despite no formal requests for participation, or marketing of the survey. Over three quarters of participants reported a high level of satisfaction with SMBG, as indicated by a score of 7 or above on a scale of 1-10 assessing helpfulness of SMBG, with 41% scoring 10.

Despite this, there were mixed results from this group of SMBG users. For example, most participants (84%) reported having no concerns about SMBG, yet worry and anxiety were frequently reported in the free text responses. The dischord between questionaire answers and free text regarding out of range readings is curious. It could simply be that individuals perceived the question to be associated with the technicalities of using the device rather than interpreting the results. Or it could be that the reported increased anxiety is more to do with having Type 2 diabetes than the mechanism of self-monitoring of blood glucose. Furthermore, when readings are high and anxiety is increased, this emotion may encourage a person to act in response to that high reading.

It could be speculated that individuals newly diagnosed with Type 2 diabetes might be more likely to adhere to healthcare professional advice regarding testing frequency and what action to take based on the results, but this was not supported in the results.

These results suggest that individuals are testing predominantly to evaluate the effect of a behaviour or dietary change, with few (5.6%) using SMBG to alter their diabetes medication. The latter finding may be because few individuals are encouraged to alter their oral glucose-lowering medication, with most leaving decisions on medication to their healthcare professionals.

The only gender difference was that. women were more likely to engage in self-chastisement than men (p = < 0.001) and to report feelings of guilt associated out of range readings. This finding reflects the qualitative data presented by Clar and colleagues (2010) which suggested that women tended to blame themselves for out of range readings and reported higher levels of self-chastisement. Men tended to rationalize such readings. Previous SMBG literature has not explored gender differences in increased anxiety/depression [4]

Limitations of the study include not having asked participants about their HbA1c, which could have been used to identify associations between frequency of testing, levels of anxiety and biomedical control. There is evidence to show, however, that the majority of people with Type 2 diabetes do not know what their Hb1c result is with only 2.9% being able to accurately recall it[5].

Very few participants reported discussing these results with their healthcare professional, although this does not necessarily mean that they did not, simply that they did not report doing so.

Limitations of the study include not having asked participants about their HbA1c, which could have been used to identify associations between frequency of testing, levels of anxiety and biomedical control. We expected respondents to be confirmed users and supporters of SMBG, but another characteristic, given that the survey was not advertised, must have been that they are regular visitors to the Diabetes UK website. They may therefore be a group of highly motivated "expert" users. That raises implications for any increase in use of SMBG, with one question being how best to motivate others.

Other weaknesses of this survey are that it did not try to link use of SMBG with outcomes. In retrospect, we might have asked about frequency and severity of hypoglycaemic episodes; education received; and whether that included structured education. However, it is probable that this group consists mainly of effective self-educators, so it might make no difference whether or not they have attended a structured education course.

Nor did we ask whether the nurses involved were practice nurses or diabetes specialist nurses, nor whether diabetes care was provided mainly or solely in primary care. In retrospect, we should also have asked for more detail on oral drugs, and whether patients were on monotherapy or combination therapy. Our main interest in treatment reflected the remit of the DH working group, and our aim was to exclude insulin-users.

### Research needs

It would be useful to see if frequent users had lower HbA1c, and the effect of HbA1c results on anxiety. Is anxiety more prevalent if SMBG users know that control is unsatisfactory?

A key question is what distinguishes "effective patients". If we have a group of patients who are self-motivated and who take control of their diabetes and its management, what are their characteristics and how can we motivate others to do likewise? Should structured education for type 2 diabetes emphasise diet and physical activity more, and link SMBG with changes in lifestyle, so that beneficial changes give positive feedback via SMBG results?

The lack of any association between frequency of testing, and perceived helpfulness, was curious, and may reflect different aims of testing. Some may be testing more often than necessary. Several observational studies have reported on SMBG frequency and HbA1c and SMBG use and outcomes [6-8] however there remains inconsistency of reported benefit and/or effect.

Research is needed into whether more education on how to interpret and act on out of range blood glucose readings would improve control and reduce anxiety, and on the optimal frequency of such education.

## Conclusion

Our aim was to explore why some people with diabetes are convinced that SMBG is helpful, in contrast to the results of most of the trials. Over 80% of respondents report high satisfaction with the usefulness of SMBG and report feeling more 'in control' of their diabetes management using it. They are the satisfied users. However a minority of users report feelings of anxiety and depression and may be testing because they feel they ought to, without achieving net benefit.

While recent cost-effectiveness analysis concludes that SMBG is not cost-effective [9,1], nevertheless this may not apply in selected groups of patients who make more use of the data from SMBG. They may be gaining sufficient benefit to make SMBG cost-effective, but quality of life/utility measurement is needed to confirm this.

## Abbreviations

GP: General Practitioner; HbA1c: Haemaglobin A1c; HCP: Healthcare Professional; SMBG: Self Monitoring of Blood Glucose

## Competing interests

The authors declare that they have no competing interests.

## Authors' contributions

KB wrote the first draft of the paper and led the writing process. AJ and KB analysed the data, NW contributed to study design and provided input into the revisions of the paper. All authors were involved in the research design or analysis and approval of final draft submitted.

## Appendix One: Copy of Questionnaire

SMBG questions for the website.

Thank you very much for taking the time to complete our survey. Your answers are extremely valuable and will help to inform ongoing work relating to self-monitoring blood glucose (SMBG) in people with Type 2 diabetes like yourself.

The survey is particularly aimed at people with Type 2 diabetes, who are not treated with insulin, to try and find out how people use SMBG and the impact it has on their day-to-day diabetes management.

Please be aware that the survey should take approximately 10 minutes. It would be very helpful if you could try to answer as many questions as possible.

1. Are you...

Male?

Female?

2. Do you have Type 2 diabetes?

Yes

No

If you answered **Yes**, go to question 3.

If you answered **No **please do not continue with the survey, thank you for your time.

3. How long has it been since you were diagnosed with Type 2 diabetes?

0-6 months

Between 6 months and 1 year

Between 1 and 3 years

Between 3 and 5 years

More than 5 years

4a. Is your diabetes treated with insulin?

Yes

No

If you answered **Yes**, please go to question 4b

If you answered **No**, please go to question 5

4b. Is your diabetes treated with insulin and other medications?

Yes

No

If you answered **Yes**, please do not continue with the survey, thank you very much for your time.

If you answered **No**, please go to question 5

5. Do you monitor your blood glucose levels using your own meter?

Yes

No

If yes go to question 6

If no, could you please say why you don't monitor your blood glucose levels?

- I monitor my glucose levels using urine testing

- I have not been advised to do so

- Other reason, please specify:

6. Typically how often do you monitor your blood glucose levels (please

tick all that apply)?

Once a day

Twice a day

Three times a day

Four times a day

More than four times a day, please state how many times a day:

Once a week

More than once a week

Fortnightly

Less than once a month

Occasional periods - monitoring frequently for a few days

Don't know

Other, please specify:

7. Typically when do you test your blood glucose levels?

(please tick all relevant responses)

Before meals

2 hours after meals

Before going to bed

During episode(s) of illness

Before I set off to drive

If I feel that I am having a hypoglycaemic episode

If I feel my blood glucose level is higher than it should be

Before I start any physical activity

During physical activity

After I have finished any physical activity

A change in my treatment is being considered

To monitor the effect of certain foods

Other, please specify:

8. Which of the following if any, do you use your blood glucose test results

to do? (please tick all relevant responses):

to monitor my health as I have recently been diagnosed with diabetes

to make adjustments to my food intake

when assessing the impact of physical activity

to confirm if I'm having a hypoglycaemic episode

to confirm if I have a high blood glucose level

to alter my diabetes medication

to inform me about the effect of a new diabetes medication I've started

to inform me about my blood glucose levels during a period of illness

I don't use my blood glucose test result(s) to do anything

to monitor my blood glucose levels as I am trying to conceive

to monitor my blood glucose levels as I'm pregnant

to determine whether it is safe to drive

Don't know

Other, please specify:

9. If you get a high blood glucose result, for example above 10 millimoles

per litre (mmols/l), what do you do to respond to that reading?

10. If you get a low blood glucose result(s), for example below 4 millimoles

per litre (mmols/l) result, how do you deal with it?

11. Do you have any concerns/worries about self monitoring your blood glucose levels?

Yes

No

Don't know

If you answered Yes, what are your concerns?

12. On a scale of 1 to 10, with 1 meaning extremely unhelpful and 10 meaning extremely helpful, how helpful or unhelpful do you find self-monitoring blood glucose (SMBG)?

1..2..3..4..5..6..7..8..9..10

If you answered 7 or above, please say why you find it so helpful (tick all that apply below and any additional reasons):

- improved my diabetes control

- weight control

- helped to avoid episodes of hypoglycaemia

- better able to undertake physical activity

- helped me during an episode of illness

- other (please specify)

If you answered 4 or below, please say why you don't find it helpful (please list all of your reasons):

- it is painful

- my healthcare team doesn't look at the results

- it is a reminder of illness

- sometimes, I just don't want to self-monitor blood glucose

- it discouraged me from making any changes to my lifestyle

- other

13. How does it make you feel if your blood glucose levels were not what you expected them to be?

14. If your blood glucose levels were not what you expected, which of the following actions, if any did you do?

- I adjusted my medication

- I did more physical activity

- I adjusted my carbohydrate for the same mealtime the next day

- I didn't do anything

- I contacted a member of my diabetes healthcare team for advice, please state

who this was:

- other, please specify:

15. Do you discuss these results with and/or receive any education from any of your diabetes healthcare team, for example nurse, pharmacist, doctor about any of the following?

- How to use your meter?

Yes

No

I don't know

If yes, who provided you with this information?

Nurse/pharmacist/doctor/other

- Information about what your own target blood glucose ranges are?

Yes

No

I don't know

If yes, who provided you with this information?

Nurse/pharmacist/doctor/other

- Advice about when to monitor your blood glucose levels?

Yes

No

I don't know

If yes, who provided you with this information?

Nurse/pharmacist/doctor/other

- Advice about how often to monitor your blood glucose levels?

Yes

No

I don't know

If yes, who provided you with this information?

Nurse/pharmacist/doctor/other

- Advice about what to do with your blood glucose results?

Yes

No

I don't know

If yes, who provided you with this information?

Nurse/pharmacist/doctor/other

- Advice about what to do if your blood glucose results were outside of your agreed target range?

Yes

No

I don't know

If yes, who provided you with this information?

Nurse/pharmacist/doctor/other

End
